# Response to Direct Selection against Drought Stress in Black Cumin (*Nigella sativa* L.)

**DOI:** 10.1155/2022/6888187

**Published:** 2022-09-05

**Authors:** Fatma Kayacetin

**Affiliations:** Kalecik Vocational School, Ankara University, Ankara 06870, Turkey

## Abstract

Central Anatolia is prone to drought with low precipitation and high evapotranspiration which influence the growth of a several crops. The selection and cultivation of drought-tolerant crops that can survive and maintain under poor climatic conditions are very important. Black cumin (*Nigella sativa* L. Umbelliferae) is famous for aromatic, medicinal, and therapeutic uses in the Mediterranean region and elsewhere. Drought stress most often delays or reduces seed germination ending up in irregular and poor crop growth. The study aimed to compare the effects of −0.1, −0.2, and −0.4 MPa of PEG 6000 osmo-priming pretreatments for 12, 24, and 36 h and two PEG 6000 post-treatments of −0.05 and −0.15 MPa along with their respective controls for 14 days as post-treatment on seed germination and seedling establishment potential of Turkish cv. Cameli. The results showed significant differences among germination index, germination stresses tolerance index, germination speed, final germination percentage, seedling vigor index, mean germination time, shoot, root and seedling length, and seedling fresh and dry weight of pretreatments and post-treatments when compared to nonprimed and hydro-primed seeds used as control treatments. Osmo-priming pretreatments of black cumin using −0.2 or −0.4 MPa PEG 6000 for 24 or 36 hours in a medium containing −0.05 MPa PEG 6000 as post-treatment showed improved germination efficiency, with increased adaptation ability.

## 1. Introduction

Central Anatolia is severely hit by with severe drought, low precipitation, and high evapotranspiration; which influence growth of a number of crops. Therefore, there is need to grow drought tolerant crops able to survive and maintain under these conditions.

Black cumin (*Nigella sativa* L. Umbelliferae) is an annual plant with origin in Eastern Mediterranean, South European, and West Asian countries and East African countries [1–3]. It is valued for aromatic, medicinal, and therapeutic characteristics. Significant concentrations of vital minerals (calcium, iron, phosphorus, and zinc) and vitamins (folic acid, niacin, pyridoxine, and thiamin) are found in black cumin [4, 5]. Moreover, black cumin seeds also possess a small but significant amount of essential oils (0.4–2.5%), and fixed oils including palmitic acid (20.4%) and linoleic acid (64.6%) [6]. Thymoquinone is an important constitutive part of volatile oils and is the main active compound that exhibits anti-inflammatory and anticancer activities [7, 8].

Most of the farmers grow black cumin for the local market or their family use. Its seed cultivation is on increase in Turkey during recent years with the production of 352 tons of seed in an area of 326 ha in 2013, which has increased to 6435 tons in an area of 8391 ha in 2021. Black cumin exports and imports in 2020 were 924.04 tons (^$^2.45 million) and 3406.23 tons (^$^2.03 million), respectively [9].

Black cumin is widely grown as a summer crop in Burdur, Usak, and Konya provinces of Turkey under rainfed conditions [9]; where drought stress and lack of water most often cause delayed nonuniform stand or poor crop growth and yield [10–13]. If timely rains do not occur, the seed germination period could prolong until the seeds get sufficient moisture. Therefore, early spring sowing is highly important to avoid the extreme heat and drought stress during summer for optimum seed yield.

Seed priming is carried out as a presowing treatment to influence seedling establishment by affecting seed metabolic pathways during germination and growth periods of plants [14]. Salinity, cold, and drought which prevent uptake from the soil negatively influence the intake of water by seeds affecting the seedling establishment [11–15].

Seed germination and fast seedling growth are the very sensitive stages [11–17] for improved black cumin production, which is mainly grown under rainfed conditions with unfavorable and no-uniform moisture contents. Priming improves essential metabolic pathways by inducing high osmotic potential during abiotic stress that prevents seeds to absorb enough water for radicle emergence by suspending the lag phase and inhibit germination [18–20]. The priming techniques are the most effective ways of reducing germination time and improving total germination percentage, to enhance yield [20]. Various seed priming methods are used to improve germination and vigor.

Osmo-priming with PEG 6000 (polyethylene glycol) is metabolically inert and able to bind water molecules, found to mimic drought stress by lowering water potential due to osmotic stress [21].

The improved effects of osmo-priming with PEG 6000 on germination in savory [22], wheat [23], sesame [24], and tomato [25], while hydro-priming on germination in sorghum [26], balm [27], and niger [28] have been previously reported.

The current study aimed to compare the effects of osmo-priming pretreatments and post-treatments nonpriming, hydro-priming on Turkish black cumin cultivar Cameli, along with nonprimed and hyro-primed seeds as control treatment to collect data to find a relationship between germination and growth parameters.

## 2. Materials and Methods

This study was carried out using certified seeds of the *Nigella sativa* cv. Cameli, obtained from the Transitional Zone Agricultural Research Institute Eskisehir, Turkey. Preweighed seeds were immersed in a 1% (v/v) sodium hypochlorite for 5 min. Thereafter, these were rinsed with double distilled water for 3 × 3 min before priming at 25 ± 1°C. Each pretreatment and post-treatment was replicated three times.

This study compared the effects of pretreatments and post-treatments of PEG and 6000 treatments with nonprimed and distilled water hydro-primed seeds were used as control treatments 1 and 2, respectively. All seeds were carefully dried after subjecting them to any type of priming treatments including hydro and osmo-priming treatments for 12, 24, and 36 h at room temperature (25 ± 3°C).

Different durations of treatment were evaluated as the main factor, and different treatments (nonprimed seeds (control treatment 1), hydro-priming (control treatment 2), −0.1, −0.2, and −0.4 MPa of PEG 6000 pretreatments were evaluated as the subplot treatments. Thereafter all of them were subjected to −0.05 and −0.15 MPa PEG 6000 post-treatments as subfactor [29].

A three factorial experimental design with three replications was used. Each treatment contained sixty seeds. These seeds were equally distributed into three replications (20 seed per replication × 3 = 60 seeds) for germinating them in 100 × 10 mm petri dishes; where the seeds were sandwiched in moistened Whatman filter paper no. 1, with 10 mL of distilled water. These petri dishes were incubated in the dark at 21 ± 1°C and the germinated seeds were counted after 7 days to measure germination vigor [30]. The seeds were considered germinated if the length of radicles exceeded 2 mm [14]. The germination index, germination stress tolerance index, speed germination, final germination percentage, seedling vigor index, and mean germination time were calculated [31].

(1) $$\begin{array}{c} \text{Germination index}\left(GI-\%\right)=\frac{\text{germination percentage in each treatment}}{\text{germination percentage in the control}}\times 100,\\ \text{Germination stress tolerance index}\left(\text{GSTI}-\%\right)=\frac{\text{promptness index of stressed seeds}\left[\left(n\;d2\times 1\right)+\left(n\;d4\times 0.75\right)+\left(n\;d6\times 0.50\right)+\left(n\;d8\times 0.25\right)\right]}{\text{promptness index of control seeds}\left[\left(n\;d2\times 1\right)+\left(n\;d4\times 0.75\right)+\left(n\;d6\times 0.50\right)+\left(n\;d8\times 0.25\right)\right]}\times 10\text{,} \end{array}$$

where PI (promptness index) = (nd2 × 1) + (nd4 × 0,75) + (nd6 × 0,50) + (nd8 × 0,25). nd2, nd4, nd6, and nd8 denote the number of seeds germinated on day 2, 4, 6, and 8, respectively [32–35].

(2) $$\begin{array}{c} \text{Speed of germination}\left(SG-\%\right)=\frac{\text{total number of germinated grains}}{\text{total number of observed grains}}\times 100,\\ \text{Germination percentage}\left(GP-\%\right)=\frac{\text{total number of germinated grains}}{\text{total number of observed grains}}\times 100,\\ \text{Seedling vigor index}\left(SVI-\%\right)=\left(\text{average radicle length}+\text{average plumule length}\right)\times \text{germination percentage,}\\ \text{Mean germination time}\left(MGT-\text{day}\right)=\frac{n1\times d1+n2\times d2+n3\times d3+\ldots }{\text{total number of days}}\times 100. \end{array}$$

**Figure 1:**
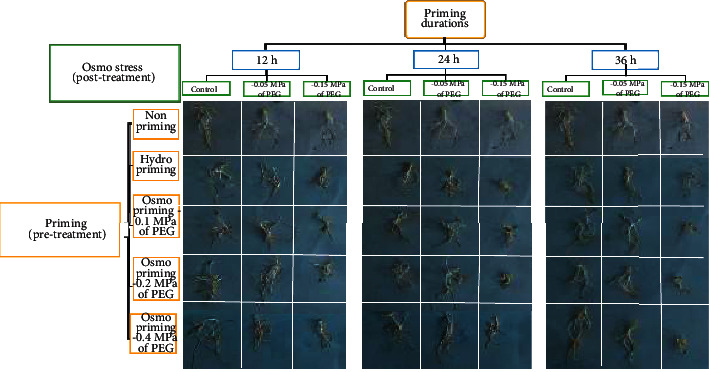
Black cumin seed germination and changes in related seedling growth parameters as noted on nonprimed, hydro-primed, and PEG-induced osmo-primed pretreated seeds for 12, 24, and 36 hours after 14 d post-treatment with 0, −0.05, and −0.15 MPa PEG.

Rest of the parameters including root length, shoot length, seedling fresh, and dry weight (Tables 1–3 and Figure 1) were measured at the end of 14^th^ day of germination [14]. The seedling dry weights were noted soon after taking fresh weights by holding the samples for 48 h at 70°C in an oven [36].

Analysis of variance (ANOVA) was computed for seed germination and growth parameters. The comparison of the treatment means was performed by LSD test (*p* < 0.05) using MSTAT-C program (Michigan State University v. 2.10).

## 3. Results and Discussion

A significant three-way interaction (*p* < 0.05) [pretreatment durations × pretreatment seed priming × postosmo priming PEG doses] was found for all germination parameters as shown in Tables 1–3 and Figure 1. The studied parameters showed positive effects of PEG-based prepriming and postpriming treatments on selection of seeds and their adaptability under stress. Control and −0.05 MPa based post PEG treatments had significantly positive effects and −0.15 MPa of PEG-based post-treatment induced significantly negative effects on the investigated parameters regardless of their treatment durations (12, 24, or 36 hours).

The maximum germination speed (19.2%) was obtained after prepriming treatment with−0.2 MPa PEG for 36 h in a medium containing 0 (control) MPa PEG as post-treatments. The lowest germination index (4.4%) was obtained after prepriming treatment with −0.2 and −0.4 MPa PEG for 36 h and post-treatment with −0.15 MPa PEG. Increasing prepriming durations significantly increased germination speed. But, increasing PEG-based extended post-treatments significantly inhibited germination speed. Osmo-priming pretreatment durations clearly improved germination speed. Sofinoris [37] reported that PEG treatments caused a reduction in air potential. It is important to find the rate of this reduction in water to determinate the reducing molecular weight of PEG. The water potential of a specific medium with PEG is utilized to simulate the magnitude of specific air potential. This is desired for reducing metabolic processes undergoing in seeds, so that nutrient reserves in endosperm are not exhausted during germination [38]. The results are in accordance with the findings of Abdallah et al. [39] for rice. Nonprimed seeds were not able to exploit the full potential of seeds during germination [40] and hydro-priming induced inhibition on seed germination [41].

The maximum mean germination time (3.2 d) was obtained with the 24 h priming pretreatment using −0.2 MPa based pretreatment with PEG and −0.15 MPa of PEG 6000 post-treatment, while the lowest mean germination time (1.0 d) was obtained using −0.2 MPa based pretreatment with PEG and in a medium containing 0 (control) as post-treatments for extended duration of 36 h. All priming durations shortened the mean germination time; however, −0.2 MPa PEG based extended post-treatment with of PEG shortened it more compared to other extended post-treatments. Mean germination period was minimized by seed priming extended post-treatments and their durations. However, any post-treatment was not favorable and delayed germination considerably. This could be explained as germination osmo-priming pretreatments which induced faster water uptake compared to the amount of water desired for seed germination. These results are not in agreement with Trisnawaty et al. [42], who found that rice seeds primed with PEG improved both germination indices and reduced the germination time.

A high germination index is desirable because it is considered as an indicator of seed strength. Considering the results, the maximum germination index (16.4%) was obtained with the pretreatment of −0.2 MPa PEG based osmo-priming and in a medium containing 0 (control) as post-treatments with 36 h priming durations, while the lowest germination index (4.6%) was obtained with the application of −0.2 MPa −0.4 MPa of PEG osmo-priming pretreatment for 36 h durations and −0.15 MPa of PEG post-treatment. Different durations of PEG pretreatments induced significantly different and positive effects on seed germination after post-treatments. Therefore, it seems that in the presence or absence of post-treatment osmo stress, effect of priming durations mattered and induced significantly different effects on germination index. Furthermore, irrespective of pre- and post-PEG priming treatments, they had improved effect on germination of seeds and seedlings growth compared to hydro-priming (control 2) [10, 43].

The lowest germination percentage (55%) was obtained with the application of 36 h duration at −0.4 MPa of PEG-based osmo-priming pretreatments after being post-treated with −0.15 MPa PEG osmo stress. Nonprimed seeds, 12 h duration × −0.1 MPa PEG prepriming treatment × hydro-primed seeds, 12 h × −0.1 MPa of PEG prepriming treatment × −0.05 MPa of post-treatment with PEG, 24 h × hydro-priming treatment for 24 h × postcontrol, 24 h × hydro-priming treatment × −0.05 MPa of PEG-based post-treatment, 36 h × pre-nonpriming treatment × control post-treatment detected the maximum germination percentages (100%) (Tables 1–3) among them. During the first germination stages, fast water uptake slows down after the seed-based metabolic activities and ends up with the emergence of radicles leading to the germination. It slowed the moving process because the seeds needed a long period to reach complete pregermination. Therefore, 36 h is considered as an optimum duration for osmo-priming (Table 3). Sadeghi et al. [44] noted that soybean seeds priming with PEG, −1.2 MPa for 12 h improved the germination percentage. Trisnawaty et al. [42] indicated the helpful effects of seed priming on rice seed germination percentage. Mirmazloum et al. [45] reported that the 5% PEG treatment with 24 h priming duration was recommended as the best treatment for improving the caraway germination percentage in comparison to nonprimed seeds and 12 h hydro-priming which was considered as the best priming duration for caraway. It was less effective in comparison to PEG pretreatment. Seeds always germinated better in higher concentrations (15%−2.5 MPa or 20%−3.9 MPa) of PEG osmo-priming than hydro-priming at the equivalent water potential in accordance with the previous reports on rice by Kartika et al. [13]. This could be due to pregermination, imbibition, and occurrence of activation phases with improved activation of physiological and metabolic activities [13].

The maximum germination stress tolerance index of the cultivar (115.69%), which was observed at 36 h prepriming time after −0.2 MPa of PEG osmo prepriming under control post-treatment, while the lowest germination index (45.59%) was obtained with the application of 36 h priming time at −0.4 MPa of PEG osmo-priming pretreatment after −0.15 MPa of PEG-based extended post-treatment stress; whereas, increasing priming durations and osmo-priming dose levels induced significant reduction in germination parameters (Table 3). Pretreatment durations and extended postpriming treatments clearly led to increasing germination stress tolerance index in this study. In addition, compared to hydro-priming, PEG-based priming pretreatments had an improved effect on germination of seeds and seedlings growth under drought conditions [10, 43].

The maximum seedling vigor index (9.1%) was obtained with the application of all priming durations using nonprimed seeds *n* in a medium containing 0 (control) MPa PEG as post-treatments, while the lowest seedling vigor index (1.6%) was obtained with the application of 36 h long −0.4 MPa of PEG priming pretreatment after extended −0.2 MPa of PEG-based post-treatment (Table 3). Primed seeds produced highly vigorous plants with rapid germination in comparison to the control treatment. In this study, seed priming pretreatment and extended post-treatment with PEG displayed as a profitable strategy for improvement of the black cumin seedling vigor index. Seedling vigor index (final germination percentage × seedling length) is an important parameter in the main seedlings establishment [46, 47] The desired effects of seed priming using PEG 6000 has a higher germination percentage and seedlings length in comparison to nonprimed seeds. Therefore, the seedling vigor index increased. Because this parameter is multiplied by the final germination percentage and seedling length or weight [48, 49]. PEG solution's high concentration inhibited water absorption and released free oxygen radical production, cell membrane damage, and changes in enzyme activity with ultimate reduction of seed germination along with seedling vigor index.

The maximum shoot length (4.6 cm) was obtained with −0.1 MPa of PEG for 36 h priming pretreatment under control post-treatment, while the minimum shoot length (0.9 cm) was obtained with the application of 36 h priming pretreatment with −0.4 MPa PEG after extended post-treatment with −0.15 MPa of PEG-based osmo stress (Table 3). The maximum root length (4.8 cm) was obtained with the application of 36 h priming pretreatment at −0.4 MPa PEG, while the minimum root length (1.5 cm) was obtained with the application of 36 h priming pr-treatment at −0.2 MPa of PEG using −0.15 MPa of PEG-based extended postosmo stress (Table 3). The maximum seedling length (9.1 cm) was obtained using nonprimed (control treatment 1), while the minimum seedling length (2.5 cm) was obtained with prepriming treatment of 36 h with −0.4 MPa of PEG using −0.15 MPa of PEG-based post-treatment. The study revealed that high drought stress (−0.15 MPa PEG osmo stress post-treatment) probably affected germination by reducing the related water uptake.

Noori et al. [46] has also reported that different seed priming methods under osmotic stress resulted in the significant improvement in seedling growth. PEG has shown to affect many aspects of plant growth, including seed germination and seedling growth [11, 14, 50]. The increase in the seedling growth parameters with prepriming and postpriming treatments could play a crucial role in regulating plant's primary and secondary seedling growth. Therefore, postgermination growth was also improved by osmo-priming seedling growth. Considering both types of seed priming, it was noted that these improved seedling growth parameters and ended up with the maximum root and shoot length. Kayacetin [11], Kayacetin [14], Trisnawaty et al. [42] found that root and shoot growth decreased significantly with increased drought stress, showing up increased length of radicles and plumules [51]. Trisnawaty et al. [42] highlighted that the longest rice seed immersion time in osmo-priming conditions was affected with priming concentrations of 100 g L^−1^ of PEG, which was around 36 hours. Priming may reduce the effects of temperature and groundwater stress, and germination times, with improved germination performance, root and shoot length [52].

The maximum seedling fresh weight (2.42 mg) was obtained with the application of 36 h priming pretreatment using −0.1 MPa of PEG priming based pretreatment and control MPa PEG as post-treatments, while the minimum seedling fresh weight (0.50 mg) was obtained with the application of 36 h priming pretreatment at −0.1 MPa of PEG osmo-priming and −0.15 MPa PEG postpriming stress (Table 3). The maximum seedling dry weight (0.23 mg) was obtained with the application of 36 h priming pre-treatment with −0.2 MPa PEG-based prepriming and −0.05 MPa PEG-based post-treatment (Table 3). While osmo-priming pretreatments significantly increased the seedling dry weight in black cumin seedlings compared to the nonprimed control treatments. The results of this study are in agreement with Kayacetin [11] and Trisnawaty et al. [42], who reported that the seedlings had the higher seedling dry weight under stress conditions. Kartika et al. [13] indicated that seeds primed with −0.1 MPa PEG improved seedling growth compared to other priming treatments. Faijunnahar et al. [53] obtained the best seedling growth when wheat seeds were treated with −0.1 MPa dose of PEG 6000 compared to nonprimed and hydroprimed seeds ending up in gradual seedling growth with decreased germination at higher PEG concentrations. The seedling growth at higher PEG concentrations was noted owing to imbalance in nutrient uptake, toxic ions mobility, and reduction of applied solutes mobility [54].

Furthermore, the results showed that treating with PEG 6000 was a successful technique to improve seed germination under stress induced by droughts. Black cumin seeds germination response was negatively affected by the PEG-induced drought stress in the present study. Drought stress influences germination indices by induction of limited water absorption, transferance of seed reserves, or directly affects the embryonic protein synthesis and organic structure [46]. Poor seed germination could be attributed to lesser water uptake under drought stress. PEG priming of black cumin seeds also improved germination parameters and seedling growth regardless of induction of stress and nonstress conditions in the present study.

## 4. Conclusions

Seeds of black cumin (cv. Cameli) were subjected to −0.1, −0.2, and −0.4 MPa of polyethylene glycol (PEG-6000) for three durations (12, 24, and 36 h) as pretreatment. These were also subjected to −0.05 and −0.15 MPa doses of PEG 6000 as post-treatment. Germination percentage, and all seed growth parameters were evaluated by comparing their performance with nonprimed seeds and hydro-primed seeds as control treatments. The results clearly indicated a reduced growth on germination and related growth parameters on nonprimed and hydro-primed seeds compared to osmo-primed seeds. This indicated that cv. Cameli could be recommended as a valuable drought tolerant genotype.

## Figures and Tables

**Table 1:** Comparison of black cumin seed germination and changes in related seedling growth parameters to nonprimed (control 1) and hydro-primed seeds (control 2) with PEG 6000-induced osmo-primed pretreated seeds for 12 h.

Priming (pretreatment)	Osmo stress (post-treatment)	Germination index (%)	Germination stress tolerance index (%)	Speed germination (%)	Final germination percentage (%)	Seedling vigor index (%)	Mean germination time (d)	Shoot length (cm)	Root length (cm)	Seedling length (cm)	Seedling fresh weight (mg)	Seedling dry weight (mg)
Nonprimed seeds	Control treatment 1	9.5	100.00	8.1	100.0	9.1	2.6	4.4	4.7	9.1	2.18	0.19
	−0.05 MPa of PEG	11.3	104.90	11.1	98.3	8.1	2.2	4.0	4.2	8.2	2.05	0.17
	−0.15 MPa of PEG	9.9	93.63	9.6	91.7	6.6	2.3	3.5	3.7	7.2	1.71	0.16

Hydro-primed seeds	Control treatment 2	10.0	98.53	9.4	96.7	8.4	2.4	4.3	4.3	8.7	1.99	0.19
	−0.05 MPa of PEG	8.1	88.24	7.1	90.0	6.0	2.7	3.5	3.1	6.6	1.67	0.16
	−0.15 MPa of PEG	4.6	54.41	4.5	65.0	3.3	3.2	2.4	2.7	5.1	1.24	0.14

−0.1 MPa of PEG	Control	12.6	106.86	13.3	100.0	8.8	1.9	4.3	4.6	8.8	1.96	0.17
	−0.05 MPa of PEG	11.2	103.92	11.0	100.0	8.4	2.2	4.3	4.1	8.4	1.98	0.18
	−0.15 MPa of PEG	7.8	76.47	7.8	80.0	4.2	2.6	2.6	2.7	5.3	1.44	0.14

−0.2 MPa of PEG	Control	14.0	107.84	15.5	96.7	8.1	1.5	4.0	4.5	8.4	2.14	0.18
	−0.05 MPa of PEG	11.7	101.96	11.9	95.0	7.3	2.0	4.0	3.7	7.7	2.10	0.20
	−0.15 MPa of PEG	8.4	82.84	8.9	90.0	3.8	2.7	2.2	2.0	4.2	1.16	0.14

−0.4 MPa of PEG	Control	10.1	99.51	9.6	98.3	7.9	2.4	3.8	4.3	8.0	1.91	0.19
	−0.05 MPa of PEG	9.3	90.69	9.4	95.0	5.4	2.5	3.1	2.5	5.7	1.31	0.17
	−0.15 MPa of PEG	5.7	64.22	5.8	75.0	3.4	3.0	2.2	2.3	4.5	1.17	0.15

LSD (*p* < 0.05)		0.3512	3.5135	0.3017	3.7232	0.2796	0.0767	0.1117	0.0635	0.1263	0.0422	0.0050

^*∗∗*^Significant at *p* < 0.01.

**Table 2:** Comparison of black cumin seed germination and changes in related seedling growth parameters to nonprimed (control 1) and hydro-primed seeds (control 2) with PEG 6000-induced osmo-primed pretreated seeds for 24 h.

Priming (pretreatment)	Osmo stress (post-treatment)	Germination index (%)	Germination stress tolerance index (%)	Speed germination (%)	Final germination percentage (%)	Seedling vigor index (%)	Mean germination time (d)	Shoot length (cm)	Root length (cm)	Seedling length (cm)	Seedling fresh weight (mg)	Seedling dry weight (mg)
Nonprimed seeds	Control treatment 1	9.5	100.98	8.1	100.0	9.1	2.6	4.4	4.7	9.1	2.18	0.19
	−0.05 MPa of PEG	11.3	104.90	11.1	98.3	8.1	2.2	4.0	4.2	8.2	2.05	0.17
	−0.15 MPa of PEG	9.9	93.63	9.6	91.7	6.6	2.3	3.5	3.7	7.2	1.71	0.16

Hydro-primed seeds	Control treatment 2	13.5	100.00	15.0	100.0	6.6	1.7	3.4	3.3	6.6	1.91	0.18
	−0.05 MPa of PEG	11.6	103.43	11.9	100.0	7.1	2.1	3.7	3.4	7.1	1.93	0.19
	−0.15 MPa of PEG	6.0	73.04	6.0	90.0	4.2	3.2	2.4	2.3	4.7	1.30	0.14

−0.1 MPa of PEG	Control	9.3	96.57	8.3	96.7	6.3	2.6	3.2	3.3	6.6	1.86	0.19
	−0.05 MPa of PEG	14.5	108.82	16.1	98.3	6.3	1.4	3.4	3.0	6.4	1.89	0.21
	−0.15 MPa of PEG	7.6	79.90	8.0	95.0	4.6	2.9	2.6	2.3	4.9	1.07	0.22

−0.2 MPa of PEG	Control	12.5	103.43	13.5	96.7	6.4	1.8	3.2	3.4	6.6	2.11	0.15
	−0.05 MPa of PEG	10.5	94.12	11.2	95.0	8.2	2.3	4.5	4.2	8.7	2.21	0.22
	−0.15 MPa of PEG	7.0	81.37	6.7	95.0	3.9	3.0	2.0	2.1	4.1	1.36	0.18

−0.4 MPa of PEG	Control	11.2	97.55	12.0	96.7	6.3	2.2	3.3	3.3	6.6	1.71	0.17
	−0.05 MPa of PEG	9.1	91.18	9.0	95.0	7.7	2.6	4.2	4.0	8.1	1.94	0.21
	−0.15 MPa of PEG	7.5	81.86	7.8	95.0	3.8	3.0	1.9	2.1	4.0	1.13	0.18

LSD (*p* < 0.05)		0.3512	3.5135	0.3017	3.7232	0.2796	0.0767	0.1117	0.0635	0.1263	0.0422	0.0050

^*∗∗*^Significant at *p* < 0.01.

**Table 3:** Comparison of black cumin seed germination and changes in related seedling growth parameters to nonprimed (control 1) and hydro-primed seeds (control 2) with PEG 6000-induced osmo-primed pretreated seeds for 36 h.

Priming (pretreatment)	Osmo stress (post-treatment)	Germination index (%)	Germination stress tolerance index (%)	Speed germination (%)	Final germination percentage (%)	Seedling vigor index (%)	Mean germination time (d)	Shoot length (cm)	Root length (cm)	Seedling length (cm)	Seedling fresh weight (mg)	Seedling dry weight (mg)
Nonprimed seeds	Control treatment 1	9.5	100.00	8.1	100.0	9.1	2.6	4.4	4.7	9.1	2.18	0.19
	−0.05 MPa of PEG	11.3	104.90	11.1	98.3	8.1	2.2	4.0	4.2	8.2	2.05	0.17
	−0.15 MPa of PEG	9.9	93.63	9.6	91.7	6.6	2.3	3.5	3.7	7.2	1.71	0.16

Hydro-primed seeds	Control treatment 2	13.9	107.84	15.5	98.3	6.7	1.6	3.7	3.1	6.8	1.58	0.18
	−0.05 MPa of PEG	12.4	101.47	13.4	95.0	5.8	1.8	3.0	3.1	6.1	1.90	0.20
	−0.15 MPa of PEG	5.1	54.41	4.4	55.0	1.9	2.6	1.8	1.8	3.5	0.84	0.18

−0.1 MPa of PEG	Control	16.1	114.22	18.7	98.3	7.7	1.1	4.6	3.2	7.8	2.42	0.21
	−0.05 MPa of PEG	10.6	98.04	10.9	98.3	7.4	2.3	4.1	3.5	7.5	1.84	0.22
	−0.15 MPa of PEG	6.0	60.78	5.9	65.0	1.9	2.7	1.4	1.6	2.9	0.50	0.16

−0.2 MPa of PEG	Control	16.4	115.69	19.2	96.7	6.8	1.0	3.009	3.2	7.0	2.09	0.20
	−0.05 MPa of PEG	13.3	105.88	14.8	98.3	6.5	1.7	3.4	3.2	6.6	1.92	0.23
	−0.15 MPa of PEG	7.7	77.94	7.5	75.0	2.3	2.4	1.6	1.5	3.1	0.83	0.17

−0.4 MPa of PEG	Control	11.5	96.08	12.5	95.0	8.4	1.9	4.1	4.8	8.9	2.05	0.17
	−0.05 MPa of PEG	8.8	83.82	9.3	90.0	6.0	2.5	3.2	3.5	6.7	1.71	0.16
	−0.15 MPa of PEG	4.6	45.59	4.4	65.0	1.6	3.2	0.9	1.6	2.5	0.72	0.14

LSD (*p* < 0.05)		0.3512	3.5135	0.3017	3.7232	0.2796	0.0767	0.1117	0.0635	0.1263	0.0422	0.0050

^*∗∗*^Significant at *p* < 0.01.

## Data Availability

The data are available on request.
